# The Genome: An Outsider's View

**DOI:** 10.1371/journal.pcbi.0020156

**Published:** 2006-12-29

**Authors:** Carl Zimmer

The Buddha once told a story about a king who ordered a group of blind men to be presented with an elephant. Each man touched a different part of the animal. The king then asked them what an elephant is like.

The blind men who touched the elephant's head replied, “An elephant, your majesty, is just like a water jar.” The blind men who touched its ear said, “An elephant, your majesty, is just like a winnowing basket.” The blind men who touched its tusk declared, “An elephant, your majesty, is just like a plowshare.” The ones who touched the trunk replied, “An elephant, your majesty, is just like a plow pole.” The blind men who touched the body replied, “An elephant, your majesty, is just like a storeroom.” The blind men who touched the foot replied, “An elephant, your majesty, is just like a post.” The blind men who touched the hindquarters replied, “An elephant, your majesty, is just like a mortar.” The blind men who touched the tail replied, “An elephant, your majesty, is just like a pestle.” And the blind men who touched the tuft at the end of the tail replied, “An elephant, your majesty, is just like a broom.”

The blind men fell into a fistfight, shouting, “An elephant is like this, an elephant is not like that! An elephant is not like this, an elephant is like that!” [1]

I am a science writer, and my chief passion is biology. I spend time with biologists of all stripes—computational biologists, paleontologists, biochemists, ecologists, and all the rest. It is a marvelous privilege. But there are times, I must confess, when I feel like I am watching a blind fistfight.

One of the first bouts I witnessed took place in the late 1990s, when I was researching the origin of whales. Whales descend from terrestrial mammals, and made the transition to water between about 50 million and 40 million years ago. In the 1990s paleontologists began unearthing a series of spectacular fossils documenting that transition, including whales with full-blown legs. Functional morphologists joined forces to investigate the transition, studying swimming otters to understand how proto-whales might have moved through the water. I spent a lot of time with scientists such as these. They are naturalists. They have to know a lot of natural history. They have to memorize the details of many species, to understand how the physiology, behavior, morphology, and ecology of each animal add up into an integrated whole. Yet these naturalists also know that they only have a slippery grasp on all of that embodied complexity.

I put what I learned from those naturalists into my first book, *At the Water's Edge* [2]. As I was finishing up my manuscript, I began coming across papers in which scientists were taking a radically different approach to the question of whale origins: they were comparing the DNA of whales to that of other mammals.

At the time, molecular phylogenies were still a novelty. The computational methods for calculating them were relatively new, and scientists could only use them to compare a few gene sequences of whales and other mammals. But the conclusions from these few studies were the same: the closest living cousins of whales are hippos.

This may not seem like a big deal. It certainly didn't seem to bother the scientists who carried out the studies. They were just analyzing digital code, abstracted from the animals that carried it. The results spoke for themselves. Nevertheless, they gave paleontologists and mammalian systematists conniptions.

Here's why. Hippos are artiodactyls (also known as even-toed ungulates). Other artiodactyls include cows, camels, and pigs, and goats. Zoologists have long recognized a number of anatomical features that unite artiodactyls in their own group, distinct from other hoofed mammals such as horses and rhinos. One of the most obvious hallmarks was a bone in the ankle, the astragalus. The artiodactyl astragalus has a unique double-pulley shape that allows the artiodactyl hoof to swing back and forth in a distinctive way.

In the 1990s paleontologists found a number of spectacular early whale fossils, but they had yet to find bones from the whale ankle. The bones and teeth they did find suggested that the closest relatives of whales were an extinct group of mammals called mesonychids. Mesonychids were hoofed mammals, but they did not have a double-pulley astragalus. Therefore, paleontologists concluded, they were probably not artiodactyls. And if mesonychids were not artiodactyls, then whales could not be either. And that meant that the whale–hippo link had to be wrong.

As far as I could tell, this line of reasoning caused no distress among the scientists who found the hippo–whale link in their genetic data. Frankly, I'm not sure they knew what an astragalus was. How many computational biologists are trained in anatomy? The shape of the mesonychid astragulus was irrelevant to them, really. To them, DNA was an overwhelmingly superior source of information. It could be analyzed precisely. It was strings of code, rather than a maddening blur of phenotype.

I decided that this potential whale–hippo link was important enough to mention in my book, but did so only briefly because the results were so preliminary. Still, I left the matter open, and I'm glad I did. Subsequent studies on mammal DNA continued to support the whale–hippo link. And meanwhile paleontologists discovered more fossils of ancient walking whales. In 2001 Phil Gingerich and his colleagues from the University of Michigan and from Pakistan described a skeleton of the seal-like species *Rodhocetus*. Among its bones, they found an astragulus—the first whale astragulus ever discovered. And lo and behold, the astragalus had a double-pulley shape [3]. The DNA, it seems, had been right all along.

This experience made a strong impression on me. I was struck by the divide between these two kinds of biologists. Each group had a profound confidence in their own sources of information, and an abiding skepticism about the other's. As I learned more about the history of modern biology, I realized that this rift did not begin in the 1990s. It was already present in the 1950s, as molecular biologists began championing their new science over more traditional ways of understanding life.

Harvard University's biology department was a microcosm of this conflict. James Watson, fresh from discovering the structure of DNA, breezed into the department in 1956 with revolution on his mind. “It was time to sweep beyond mere description of animals and plants and move into a new biology based on chemistry and physics,” as Watson's biographer, Victor McElheny, writes [4].

Needless to say, the Harvard naturalists were not happy. Edward O. Wilson, entomologist, ecologist, and sociobiologist, pushed back hard. “Watson, having risen to historic fame at an early age, became the Caligula of biology,” he writes in his autobiography, *Naturalist*. “It was foolish, we argued, to ignore principles and methodologies distinctive to the organism, population, and ecosystem, while waiting for a still formless and unproved molecular future” [5]. The struggle only ended when Harvard's biologists agreed to split their department in two.

Over the past fifty years, these factions of biologists have had a complex relationship. Some scientists have continued to carry out relatively traditional natural history work, with little need to delve into molecular (or computational) biology. Others have given little attention to natural history, focusing their efforts instead on deciphering the complexities of a membrane channel, or building new algorithms for identifying open reading frames. In some cases, biologists have bridged this divide, and the result has been a fruitful collaboration. But in other cases—such as the DNA studies on whales and hippos—one group moves into the other's traditional territory, sparking new conflict.

“That can't be right” is a refrain I've often heard when computational biologists report a controversial result. Along with the whale–hippo link, I can also recall the snorts that came with the first studies on human DNA suggesting that we can trace our ancestry to Africa 150,000 years ago. The picture is certainly more complex than the first “out of Africa” scenarios suggested, but the core of that idea has withstood further scrutiny. These days I sense a growing confidence in computational biologists, perhaps brought on by successes such as these. Many are no longer content merely to present an analysis of DNA and leave it at that. They use their findings to make sweeping statements about ecology and evolution.

One example of this new ambition was a paper published earlier this year on the evolution of cats [6]. The scientists offered a sweeping scenario for cat evolution, complete with migrations of cats out of Asia into the New World and back, along with the emergence of the major groups of felids, ranging from ocelots to bobcats to lions. The scientists based their scenario entirely on an analysis of cat DNA. They did not consider a single fossil of a cat, nor did they have a paleontologist expert on cats as a coauthor. Cat fossil experts inform me that fossils of true cats as old as 17 million years have been discovered in North America. The geneticists put the arrival of cats in North America at only 8 million years ago. Whether or not the DNA results are correct, it is striking that the report does not even mention the existence of fossils that do not fit the pattern.

Genomes unquestionably contain a rich store of information, but sometimes that information can be deceptive. Recently, two paleontologists, Philip Donoghue of the University of Bristol and Mark Purnell of the University of Leicester, described how this deception can arise in the study of vertebrate evolution [7]. A number of studies have suggested that vertebrates underwent several rounds of genome duplication since they diverged from their closest invertebrate ancestors. One of these duplications may have coincided with the emergence of jawed vertebrates (gnathostomes). This link has prompted some scientists to propose that this duplication triggered a leap in complexity that set gnathostomes apart from their closest vertebrate relatives, the hagfish and lampreys. Gnathostomes not only have jaws, but also have a long list of other traits that set them apart from agnathans, including an advanced immune system, a mineralized skeleton, teeth, and fins. They are also far more diverse. Perhaps a genome duplication provided the raw material for an adaptive radiation.

As Donoghue and Purnell demonstrate, this idea only makes sense if you ignore the fossil record. Paleontologists have found a rich record of fossils documenting the gradual assembly of the gnathostome body plan. Conodonts, for example, possessed a mineralized skeleton, dentine, and enamel—but nothing else that sets gnathostomes apart from agnathans. Fins evolved later, and a mineralized braincase came later still. Nowhere along the phylogeny of vertebrates is there a sudden leap of complexity in the forerunners of true gnathostomes, nor is there an abrupt leap in levels of diversity. One is left to wonder just when the alleged effects of a genome duplication occurred.

Taking natural history seriously is good not only because it can steer a computational biologist away from these sorts of errors. It also opens up fascinating new questions to which computational biologists can profitably apply their skills. Consider the coelacanth, a rare, ugly fish found in the Indian Ocean. It belongs to the lobe fins, a group of fish from which we and all other land vertebrates evolved. Scientists knew about the fossils of coelacanths long before they saw one alive. The fossil record of their ancestors reaches back 400 million years, but it was not until 1938 that living coelacanths were discovered off the coast of South Africa.

I present the coelacanth in the spirit of the Buddha. Is it a fish, or a just bag of digital code?

Earlier this year, computational biologists published a remarkable paper about the coelacanth in *Nature* [8]. The paper had its origins in the startling discovery that certain regions of the human genome that do not code for proteins are highly conserved. Natural selection has kept these sequences much the same in humans and in other mammals. The scientists were puzzled as to how natural selection could preserve their homology, since these sequences do not encode any protein.

The scientists decided to see how widespread these regions are among animals. Searching gene databases, they discovered related genetic sequences in the coelacanth. Despite more than 400 million years separating our ancestors, the coelacanth and human regions were 80% identical. A close examination of the coelacanth segments revealed them to be transposons—virus-like stretches of DNA that could insert new copies of themselves into the genome.

The authors conclude that these ultraconserved regions of the human genome descend from a transposon in the ancestors of lobe fins. In the tetrapod lineages, the transposons lost their ability to spread through the genome. Some became exapted for regulatory functions, and were preserved by natural selection. The others were eroded by mutations. In the coelacanth lineage, on the other hand, the transposons remained active.

As fascinating as these results are, I was also struck by how they were framed. The authors begin their paper by referring to the coelacanth as a “living fossil.” They end their paper with the conclusion that by preserving this family of transposons, the fish “acts, in a sense, as a living molecular fossil. The remaining 99.9% of its genome, as yet unsequenced, may very well hold precious traces of additional events that helped shape our own evolution.”

The coelacanth is a living fossil only insofar as scientists once thought it was extinct. But that is not the dictionary definition of a living fossil: an organism “that has remained essentially unchanged from earlier geologic times” [9]. Paleontologists have been giving the coelacanth lineage a fresh look, and they've discovered a surprising amount of morphological change [10]. Some ancient relatives of today's coelacanths were slender, eel-like creatures. Others had round, sunfish-like bodies. Today's coelacanths are probably not living fossils in an ecological sense, either. Early fossils of coelacanths are often found in sedimentary rocks formed in coastal waters. Living coelacanths, by contrast, only live deep under water.

I would question whether the coelacanth is much of a molecular living fossil, either. The new research certainly shows that coelacanths preserve information in their genome that reveals some of the history of ultraconserved regions in our genome that would otherwise be lost. But that's because their transposons are still “alive,” while our transposons lost their ability to replicate a long time ago and have become frozen in place. In that sense, *we* are the living fossils.

If one thinks about the coelacanth's natural history as well as its genome, a new set of questions arises. Transposons are prone to mutations, which can take away their ability to insert new copies in a genome. These coelacanth transposons have been replicating for 400 million years, and now probably take up a significant fraction of the coelacanth genome. And yet they still have not diverged very much in all that time. Is natural selection conserving them? Is natural selection acting on the transposon or the coelacanth? How do these proliferating transposons affect the physiology of the coelacanth? Do they act like pathogens, or are they providing some benefit we don't yet understand? Does their expansion play any role in the ecology of living coelacanths?

None of these questions would even arise without the invaluable work of computational biologists. But if all the blind men gather together around the coelacanth, perhaps they can better understand just what this creature is. 
