# Correction: The Genome: An Outsider's View

**DOI:** 10.1371/journal.pcbi.0030047

**Published:** 2007-02-23

**Authors:** Carl Zimmer

In *PLoS Computational Biology,* vol 2, issue 12: doi:10.1371/journal.pcbi.0020156

In the Acknowledgments section of this Perspective, the spelling of the name of Pavel Pevzner was incorrect.

